# Antimalarial Activity of Kaempferol and Its Combination with Chloroquine in* Plasmodium berghei* Infection in Mice

**DOI:** 10.1155/2018/3912090

**Published:** 2018-12-02

**Authors:** Voravuth Somsak, Awatsada Damkaew, Pinanong Onrak

**Affiliations:** ^1^School of Allied Health Sciences, Walailak University, Nakhon Si Thammarat 80161, Thailand; ^2^Research Excellence Center for Innovation and Health Products, Walailak University, Nakhon Si Thammarat 80161, Thailand

## Abstract

The search for new antimalarial drugs has become an urgent requirement due to resistance to the available drugs and the lack of an effective vaccine. In this respect, the present study aimed to evaluate the antimalarial activity of kaempferol against* Plasmodium berghei* infection in mice as an* in vivo* model. Chronic toxicity and antimalarial activities of kaempferol alone and in combination with chloroquine were investigated in* P. berghei* ANKA infected ICR mice using standard procedures. The results showed that chronic administration of 2,000 mg/kg of kaempferol resulted in no overt signs of toxicity as well as no hepatotoxicity, nephrotoxicity, or hematotoxicity. Interestingly, kaempferol exerted significant (*P* < 0.05) chemosuppressive, chemoprophylactic, and curative activities in a dose-dependent manner. The highest antimalarial activity was found at a dose of 20 mg/kg which resulted in a significantly (*P* < 0.05) prolonged survival of infected mice. Moreover, combination treatment of chloroquine and kaempferol also presented significant (*P* < 0.05) antimalarial effects, although the effects were not significantly different from the chloroquine treated group. From the results of the present study, it can be concluded that kaempferol possesses acceptable antimalarial activities. However, further investigation should be undertaken on the mechanism responsible for the observed antimalarial activity.

## 1. Introduction

Malaria is still a public health problem and it is among the most deadly parasitic diseases around the world especially in many tropical and subtropical regions. Malaria is a disease that is transmitted by the bite of a female* Anopheles* mosquito, which is infected by a parasite of the genus* Plasmodium*. There are an estimated 212 million cases of malaria globally which leads to some 445, 000 deaths, most of which occur in African children under the age of five [1]. Although an effective vaccine is the best long-term control for malaria, the research on vaccine development is still at a preclinical stage and it is predicted that a malarial vaccine is still several years away [2]. However, the emergence of* Plasmodium* parasite resistance to existing antimalarial drugs, as well as* Anopheles* mosquito resistance to insecticides could render some of the current management tools ineffective and trigger a new rise in malaria mortality [3]. Hence, it is necessary to search for new, safe, and affordable antimalarial drugs for the treatment of the disease. In this respect, medicinal plants are potential resources in the search for antimalarial agents. This is because most of these plants are rich in secondary metabolites such as flavonoids, terpenoids, alkaloids, and quercetin that have been reported to have antimalarial activity [4].

Kaempferol is a natural flavonoid mostly found in tea, broccoli, apples, strawberries, and beans. It has been reported and hypothesized to be an active compound that has several activities including antioxidant, anti-inflammation, antimicrobial, antiparasite, and anticancer [5]. In addition, kaempferol has also been described to possess hepatoprotective and immunomodulatory properties [6, 7]. Previously, kaempferol isolated from the leaves of* Schima wallichii* was shown to exert a potent antimalarial activity against chloroquine-resistant* P. falciparum* [8]. However, any antimalarial activity of kaempferol against* P. berghei* and particularly in combination with chloroquine has not yet been reported. Therefore, the aim of the present study was to assess the antimalarial activity of kaempferol both alone and in combination with chloroquine using* P. berghei* infected mice as an* in vivo* model.

## 2. Materials and Methods

### 2.1. Chemicals

Chloroquine diphosphate salt (CQ) and kaempferol used in this study were purchased from Sigma (Sigma, Chemical, St. Louis, MO, US). All reagents were analytical grade and procured from certificated suppliers.

### 2.2. Drug Preparation

CQ (10 mg/kg) and kaempferol (1-2,000 mg/kg) were freshly prepared corresponding to the body weight of the experimental mouse in 0.2 ml of distilled water (DW) and orally administered by intragastric gavage.

### 2.3. Experimental Mice

Female ICR mice, 4-6 weeks of age, weighting between 25 and 35 g, were used for all experiments. The experimental mice were obtained from the National Laboratory Animal Center, Mahidol University, Thailand. They were kept at 25 ± 2°C with a 12 h photoperiod per day and provided with pellet diet and clean water* ad libitum*. All protocols were carried out in accordance with the guidelines of international animal care and welfare. All the animal experiments were approved by the Animal Ethic Committee of Walailak University (WU006/2018).

### 2.4. Chronic Oral Toxicity Assay

The chronic oral toxicity of kaempferol was evaluated according to the procedure guidelines of the Organization for Economic Co-Operation and Development (OECD) [9]. Two groups of naïve ICR mice (3 mice per group) were used. One group was administered with 2,000 mg/kg of kaempferol, while the second group received distilled water (DW) as a control, daily for 30 consecutive days. The mice were observed for signs of toxicity which included (but were not limited to) salivation, paw licking, weakness, stretching of the entire body, respiratory distress, coma, and death in the first three hours, and subsequently daily for 30 days. For evaluation of the effect of kaempferol on mouse hematological and serum parameters, mouse blood was collected by cardiac puncture into heparinized vacuum tubes on day 31 after treatment. The complete blood count (CBC) and serum parameters for blood chemistry including aspartate aminotransferase (AST), alanine aminotransferase (ALT), blood urea nitrogen (BUN), and creatinine were measured using automated analyzers (IDXX ProCyte Dx and Cobas c311, respectively).

### 2.5. Plasmodium berghei

Chloroquine-sensitive* Plasmodium berghei* ANKA strain (PbANKA) was used for the induction of malaria in the experimental ICR mice. This parasite can be obtained from the Malaria Research and Reference Reagent Resource Center (MR4; https://www.beiresources.org/Home.aspx). Mice infected with PbANKA were used as the donor source. The parasites were subsequently maintained by serial passage of blood from the donor infected mice to naïve mice via intraperitoneal (IP) injection on a weekly basis. The donor infected mice with a parasitemia of 20-30% were sacrificed, and blood was collected by cardiac puncture into heparinized tubes. The blood was subsequently diluted with 0.9% normal saline solution (NSS) and infection undertaken by injecting 0.2 ml of the diluted blood which contained 1x10^7^ parasitized erythrocytes via IP injection. Parasitemia was monitored daily by microscopic examination of Giemsa stained thin blood smears, and parasitemia was calculated using the following formula.

(1) %  parasitemia=Number  of  parasitized  erythrocytes×100Total number of erythrocytes

### 2.6. Evaluation of Suppressive Antimalarial Activity of Kaempferol

Evaluation of the chemosuppressive antimalarial activity of kaempferol was carried out using the standard 4-day suppressive test as previously described [10]. Groups of ICR mice (3 mice per group) were inoculated with 1x10^7^ PbANKA parasitized erythrocytes by IP injection. Two hours later, mice were administered 1, 10, and 20 mg/kg kaempferol by intragastric gavage followed by further administration once a day for 4 consecutive days (day 0-3). The untreated control mice were given 10 ml/kg of DW while the positive control mice were treated with 10 mg/kg of CQ. Moreover, the combination of 10 mg/kg of CQ and 20 mg/kg of kaempferol was also investigated. At day 4, parasitemia was determined and percent inhibition was then calculated using the following formula.

(2) $$\begin{array}{c} \%\;\;inhibition=\left(\;\frac{\%\text{ parasitemia of untreated control - }\%\text{ parasitemia of treated group}}{\%\;\;parasitemia\;\;of\;\;untreated\;\;control}\;\right)\times 100 \end{array}$$

### 2.7. Evaluation of Prophylactic Antimalarial Activity of Kaempferol

Evaluation of the chemoprophylactic potential of kaempferol was performed according to the method previously described [11]. Groups of ICR mice (3 mice per group) were administered 1, 10, and 20 mg/kg kaempferol by intragastric gavage once a day for 4 consecutive days (day 0-3). Untreated control mice were given 10 ml/kg of DW while the positive control mice were treated with 10 mg/kg CQ. Moreover, the combination of 10 mg/kg of CQ and 20 mg/kg of kaempferol was also administered. At day 4, the mice were inoculated with 1x10^7^ PbANKA parasitized erythrocytes by IP injection. Seventy-two hours later, parasitemia was determined and percent inhibition was then calculated as described above.

### 2.8. Evaluation of Curative Antimalarial Activity of Kaempferol

Evaluation of curative activity of kaempferol was carried out using the method previously described [12]. Groups of ICR mice (3 mice per group) were inoculated with 1x10^7^ PbANKA parasitized erythrocytes by IP injection. Seventy-two hours later, these mice were treated with 1, 10, and 20 mg/kg kaempferol administered by intragastric gavage once a day for 4 consecutive days (days 0-3). Untreated control mice were given 10 ml/kg of DW while the positive control mice were treated with 10 mg/kg of CQ. Moreover, the combination of 10 mg/kg of CQ and 20 mg/kg of kaempferol was also investigated. At day 4, parasitemia was determined and percent inhibition was then calculated as described above.

### 2.9. Determination of Mean Survival Time

The mortality of the experimental mice was monitored daily and the number of days from the time of infection up to death for each mouse in the treatment and control groups throughout the follow-up period was recorded. Mean survival time (MST) was then calculated for each group using the following formula.

(3) $$\begin{array}{c} MST=\frac{\text{Sum of survival time of all mice in a group}}{\text{Total number of mice in that group}} \end{array}$$

### 2.10. Statistical Analysis

Statistical analysis of the data was carried out using GraphPad Prism (GraphPad Prism software, Inc., US). The results are presented as mean ± standard error of mean (SEM). The differences between means of the measured parameters were compared using one-way ANOVA followed by Tukey's post hoc test. The* P* values < 0.05 at 95% confidence were regarded as statistically significant.

## 3. Results

### 3.1. Chronic Oral Toxicity of Kaempferol in Mice

Kaempferol was well-tolerated by mice over a period of administration of 30 days. Chronic administration of 2,000 mg/kg of kaempferol did not result in any overt signs of toxicity. Hepatotoxicity (indicated by AST and ALT), nephrotoxicity (indicated by BUN and creatinine), and hematotoxicity were also ruled out due to a lack of a significant difference with values from control mice (Table 1).

### 3.2. Suppressive Antimalarial Activity of Kaempferol against Plasmodium berghei ANKA

**Figure 1:**
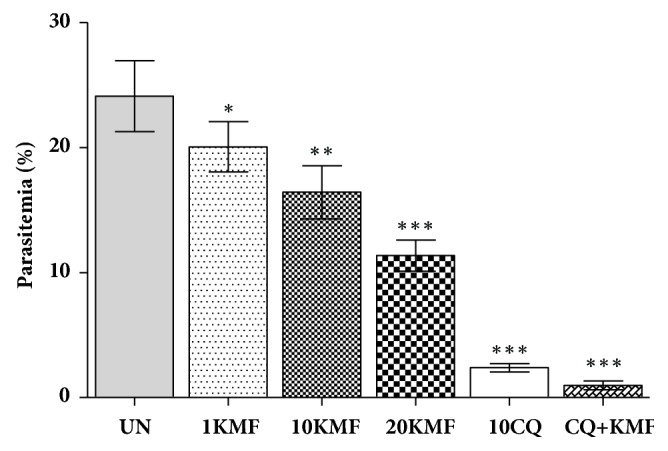
Antimalarial effect of kaempferol on PbANKA in a suppressive test. Groups of ICR mice (3 mice per group) were inoculated with 1x10^7^ parasitized erythrocytes of PbANKA by IP injection. Two hours later, they were administered with 1, 10, and 20 mg/kg of kaempferol by oral gavage. Untreated control mice received 10 ml/kg of DW while positive control mice were given 10 mg/kg of CQ. A combination treatment of 10 mg/kg of CQ and 20 mg/kg of kaempferol was also administered. The treatment was carried out for 4 consecutive days (days 0-3). On day 4, parasitemia was determined by microscopic examination of Giemsa stained thin blood smears. *∗ P* < 0.05, *∗∗ P* < 0.01, and *∗∗∗ P* < 0.001, compared to untreated control. UN; untreated control, 1KMF; 1 mg/kg of kaempferol, 10KMF; 10 mg/kg of kaempferol, 20KMF; 20 mg/kg of kaempferol, and 10CQ; 10 mg/kg of chloroquine. The results are expressed as mean ± SEM.

Kaempferol exerted a significant (*P* < 0.05) dose-dependent chemosuppression against PbANKA (Figure 1). The percentage inhibition at 1, 10, and 20 mg/kg of kaempferol was 16.79%, 31.87%, and 52.89%, respectively. The standard drug, CQ, showed a significant (*P* < 0.001) chemosuppressive effect with 90.11% inhibition, which was higher than the suppression in the kaempferol treated animals. Moreover, a significant (*P* < 0.001) chemosuppression was also observed in a combination treatment of CQ and kaempferol, but the suppression was not statistically different from CQ treated group.

### 3.3. Prophylactic Antimalarial Activity of Kaempferol against Plasmodium berghei ANKA

**Figure 2:**
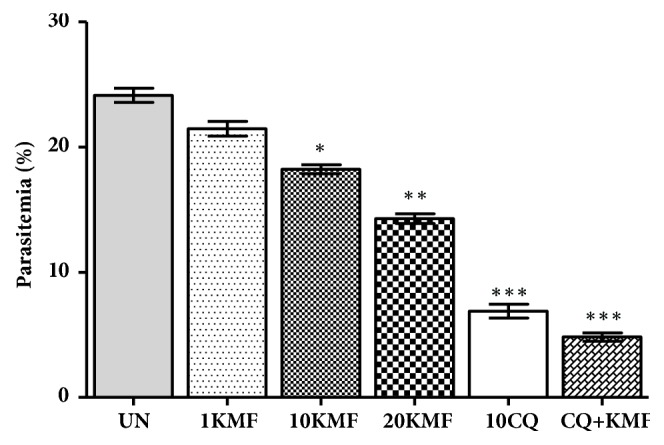
Antimalarial effect of kaempferol on PbANKA in a prophylactic test. Groups of ICR mice (3 mice per group) were given 1, 10, and 20 mg/kg of kaempferol by oral gavage for 4 consecutive days (days 0-3). Untreated and positive control mice received 10 ml/kg of DW and 10 mg/kg of CQ, respectively. A combination treatment with 10 mg/kg of CQ and 20 mg/kg of kaempferol was also undertaken. Starting on day 4, the mice were inoculated with 1x10^7^ PbANKA parasitized erythrocytes by IP injection for 4 days (days 4-7). On day 8, parasitemia was determined by microscopic examination of Giemsa stained thin blood smears. *∗ P* < 0.05, *∗∗ P* < 0.01, and *∗∗∗ P* < 0.001, compared to untreated control. UN; untreated control, 1KMF; 1 mg/kg of kaempferol, 10KMF; 10 mg/kg of kaempferol, 20KMF; 20 mg/kg of kaempferol, and 10CQ; 10 mg/kg of chloroquine. The results are expressed as mean ± SEM.

To determine the efficacy of kaempferol as an antimalarial prophylactic, mice were administered 1, 10, and 20 mg/kg of kaempferol for 4 consecutive days, while control mice received 10 ml/kg of DW or 10 mg/kg of CQ. A combination treatment was also performed with 10 mg/kg of CQ and 20 mg/kg of kaempferol. Subsequently, these mice were inoculated with PbANKA parasitized erythrocytes of PbANKA by IP injection for 4 days. On day 8, parasitemia was determined using microscopic examination of Giemsa stained thin blood smears. Results (Figure 2) showed that kaempferol possessed significant (*P* < 0.05) chemoprophylactic activity of 24.47% and 40.80% at the doses of 10 and 20 mg/kg, respectively, while the CQ treated showed significant (*P* < 0.001) chemoprophylactic effect with 71.45% inhibition. Moreover, 80.01% inhibition was found in the combination treatment of CQ and kaempferol. However, there was no statistically significant difference between the CQ treated mice with and without kaempferol.

### 3.4. Curative Antimalarial Activity of Kaempferol against Plasmodium berghei ANKA

**Figure 3:**
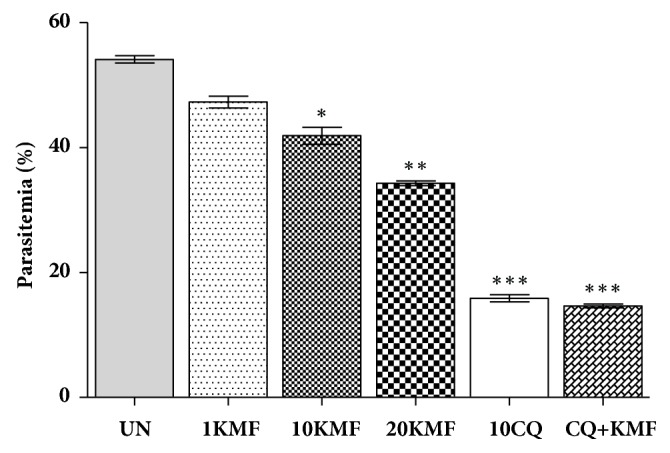
Antimalarial effect of kaempferol on PbANKA in a curative test. Groups of ICR mice (3 mice per group) were inoculated with 1x10^7^ PbANKA parasitized erythrocytes by IP injection for 4 days (days 0-3). Starting on day 4, the mice were given 1, 10, and 20 mg/kg of kaempferol by oral gavage for 4 consecutive days (day 4-7). Untreated and positive control mice received 10 ml/kg of DW and 10 mg/kg of CQ, respectively. A combination treatment of 10 mg/kg of CQ and 20 mg/kg of kaempferol was also administered. On day 8, parasitemia was determined by microscopic examination of Giemsa stained thin blood smears. *∗ P* < 0.05, *∗∗ P* < 0.01, and *∗∗∗ P* < 0.001, compared to untreated control. UN; untreated control, 1KMF; 1 mg/kg of kaempferol, 10KMF; 10 mg/kg of kaempferol, 20KMF; 20 mg/kg of kaempferol, and 10CQ; 10 mg/kg of chloroquine. The results are expressed as mean ± SEM.

To assess the curative efficacy of kaempferol mice were inoculated with PbANKA parasitized erythrocytes for 4 consecutive days. On day 4 and for the following 3 days, mice were administered 1, 10, and 20 mg/kg of kaempferol. Control mice received either 10 ml/kg of DW or 10 mg/kg of CQ. A combination treatment was also performed with 10 mg/kg of CQ and 20 mg/kg of kaempferol. Parasitemia was determined on day 8 by examination of Giemsa stained thin blood smears. Results (Figure 3) showed that kaempferol treatment resulted in a significant (*P* < 0.05) reduction in parasitemia, with an inhibition of 22.59% and 36.63% at doses of 10 and 20 mg/kg, respectively. The CQ treated group showed a significant (*P* < 0.001) reduction of parasitemia with 70.64% inhibition, which was similar to the level of inhibition seen in the combination treated mice (72.92%).

### 3.5. Effect of Kaempferol on Mean Survival Time of Mice

The efficacy of kaempferol in mice at all doses, except that of 1 mg/kg, was correlated significantly (*P* < 0.05) with increased mean survival time (MST) compared to the untreated control animals (Table 2). The MST of the CQ treated and combination treated groups were also significantly (*P* < 0.001) increased compared to untreated control animals. In the combination treatment, however, the MST was not significantly different from the CQ treated group.

## 4. Discussion

The antimalarial activities of kaempferol, both alone and in combination with CQ against PbANKA infection in mice in suppressive, prophylactic, and curative tests, were investigated. Assessment of the chronic toxicity of kaempferol showed no visible signs of toxicity, hepatotoxicity, nephrotoxicity, hematotoxicity, or death of the mice after oral administration of 2,000 mg/kg of kaempferol daily for 30 days. Hence, the kaempferol can be considered safe according to the OECD guideline which recommends a maximum dose of 2,000 mg/kg for assessing toxicity [13]. The results also showed that kaempferol significantly inhibited PbANKA growth in a dose-dependent manner. The highest activity was observed at a dose of 20 mg/kg with percent inhibition of 52.89%, 40.80%, and 36.63% for chemosuppressive, chemoprophylactic, and curative experiments, respectively. The standard 4-day test is a standard test commonly used for antimalarial screening* in vivo* in which ≥30% inhibition in parasitemia following treatment makes a compound to be considered active [14]. The present finding is in agreement with previous research investigating the use of kaempferol for malaria treatment [15]. It has also been reported that kaempferol shows antimalarial activity against chloroquine-resistant* P. falciparum* after 24 h treatment with an IC50 of 106 *μ*M [8]. Therefore, the antimalarial activity of kaempferol might be primarily dependent on its antioxidant and anticancer activities. Oxidative stress resulting from the formation of reactive oxygen species (ROS) plays a critical role in malaria infection. During propagation of the malaria parasite in erythrocytes, toxic by-products that cause hemoglobin degradation are produced [16]. Additionally, ROS and oxidative stress are also generated by activated monocytes and neutrophils during infection [17, 18]. It has also been shown that the malaria parasite causes cell membrane injury to both infected and uninfected erythrocytes through lipid peroxidation [19, 20]. As a flavonoid, kaempferol is able to inhibit oxidative stress, ROS formation, and lipid peroxidation [21–23], and there is a correlation between its antioxidant and antimalarial activities [4]. In addition, inhibition of glycogen synthase kinase-3*β* (GSK3*β*) of the malaria parasite has been reported to be a molecular basis of the antimalarial effect of kaempferol [24]. GSK3 has been described to play a critical role in the host response to malaria infection [25]. Therefore, the antimalarial effects of kaempferol in the present study might be mediated through the inhibition of GSK3*β*.

Kaempferol has been described as having anticancer properties by inducing apoptosis through the caspase cascade and the MAPK pathway [26–28], which is similar to artemisinin, an effective antimalarial drug currently in use, that also has been reported to have anticancer and related potent antimalarial activities [29]. Hence, it can be hypothesized that the antioxidant and anticancer properties of kaempferol may be responsible for the antimalarial activity observed in the present study.

MST is another parameter for evaluating the antimalarial activity of test compounds. Accordingly, test compounds that result in survival time greater than that of the untreated group are considered as active [30, 31]. This study showed that infected mice treated with 10 and 20 mg/kg of kaempferol lived significantly longer than animals in the untreated group. However, the sample sizes were small; it does allow researchers to focus on really big effect sizes. These findings provide a basis for further investigations of kaempferol as a potential candidate compound for antimalarial drug development.

## 5. Conclusions

Oral administration of kaempferol at the highest dose of 2,000 mg/kg induced no adverse effects, signifying the safety of this compound in mice. Kaempferol both alone and in combination with CQ exerted a reasonable antimalarial activity and significantly prolonged the survival time of PbANKA infected mice. A dose of 20 mg/kg of kaempferol was observed to have the strongest antimalarial activity, particularly in chemosuppression. However, these findings are only preliminary and thus further studies investigating the mechanism by which kaempferol exert its antimalarial activity thereby resulting in prolonged MST in PbANKA infected mice are recommended.

## Figures and Tables

**Table 1:** Chronic oral toxicity of kaempferol in mice.

**Parameter**	**Control (10 ml/kg of DW)**	**2,000 mg/kg of KMF**
***Liver function test***		
AST (U/L)	39.07 ± 0.42	35.23 ± 0.72
ALT (U/L)	13.75 ± 0.32	13.25 ± 0.22

***Renal function test***		
BUN (mg/dl)	45.55 ± 7.12	40.42 ± 2.34
Creatinine (mg/dl)	1.74 ± 0.31	1.57 ± 0.82

***Complete blood cells***		
Erythrocyte count (cells/mm3)	6.13x10^6^ ± 5.43 x 10^5^	6.23x10^6^ ± 5.23x10^5^
Leucocyte count (cell/mm3)	1,618 ± 43.21	1,621 ± 25.67
Neutrophils (%)	35.45 ± 0.61	37.74 ± 0.85
Eosinophils (%)	2.14 ± 0.58	3.25 ± 0.45
Basophils (%)	4.10 ± 0.36	4.01 ± 0.55
Monocytes (%)	6.99 ± 0.35	6.56 ± 0.24
Lymphocytes (%)	56.00 ± 1.47	56.67 ± 2.47

**Table 2:** Effect of kaempferol on MST of PbANKA infected mice in suppressive, prophylactic, and curative tests.

**Tests**	**Treatments**	**Doses**	**Parasitemia**	**Inhibition**	**MST**
			**(**%**)**	**(**%**)**	**(days)**
Suppressive	DW	10 ml/kg	24.12	0	9.8 ± 1.5
	Kaempferol	1 mg/kg	20.07	16.79	11.3 ± 1.2
		10 mg/kg	16.43	31.87	16.2 ± 1.2*∗*
		20 mg/kg	11.36	52.89	24.8 ± 2.5*∗∗*
	CQ	10 mg/kg	2.38	90.11	33.3 ± 2.2*∗∗∗*
	CQ + kaempferol	10 mg/kg + 20 mg/kg	0.97	95.98	35.2 ± 2.1*∗∗∗*

Prophylactic	DW	10 ml/kg	24.14	0	9.5 ± 1.0
	Kaempferol	1 mg/kg	21.48	11.02	11.2 ± 2.1
		10 mg/kg	18.23	24.47	17.3 ± 1.9*∗*
		20 mg/kg	14.29	40.80	22.2 ± 2.3*∗∗*
	CQ	10 mg/kg	6.89	71.45	26.3 ± 1.5*∗∗∗*
	CQ + kaempferol	10 mg/kg + 20 mg/kg	4.83	80.01	29.2 ± 2.1*∗∗∗*

Curative	DW	10 ml/kg	54.13	0	9.3 ± 1.5
	Kaempferol	1 mg/kg	47.29	12.64	9.7 ± 1.4
		10 mg/kg	41.9	22.59	14.0 ± 1.4*∗*
		20 mg/kg	24.30	36.63	20.0 ± 1.4*∗∗*
	CQ	10 mg/kg	15.89	70.64	25.7 ± 1.6*∗∗∗*
	CQ + kaempferol	10 mg/kg + 20 mg/kg	14.66	72.92	27.2 ± 1.5*∗∗∗*

*∗*  *P* < 0.05, *∗∗*  *P* < 0.01, and *∗∗∗*  *P* < 0.001, compared to DW.

## Data Availability

The Graph data used to support the findings of this study have been deposited in the figshare (DOI: 10.6084/m9.figshare.6981866 or https://figshare.com/s/4289e1f86751ca8ffd49).

## References

[1] WHO World Health Organization, World Malaria Report. https://www.who.int/malaria/en/.

[2] White M. T., Verity R., Churcher T. S., Ghani A. C. (2015). Vaccine approaches to malaria control and elimination: Insights from mathematical models. *Vaccine*.

[3] Haldar K., Bhattacharjee S., Safeukui I. (2018). Drug resistance in Plasmodium. *Nature Reviews Microbiology*.

[4] Pan W. H., Xu X. Y., Shi N., Tsang S. W., Zhang H. J. (2018). Antimalarial Activity of Plant Metabolites. *International Journal of Molecular Sciences*.

[5] Devi K. P., Malar D. S., Nabavi S. F. (2015). Kaempferol and inflammation: From chemistry to medicine. *Pharmacological Research*.

[6] Wang M., Sun J., Jiang Z., Xie W., Zhang X. (2015). Hepatoprotective effect of kaempferol against alcoholic liver injury in mice. *American Journal of Chinese Medicine*.

[7] Martino R., Canale F., Sülsen V. (2014). A Fraction Containing Kaempferol-3,4′-dimethylether from. *Phytotherapy Research*.

[8] Barliana M. I., Suradji E. W., Abdulah R. (2014). Antiplasmodial properties of kaempferol-3-*O*-rhamnoside isolated from the leaves of *Schima wallichii* against chloroquine-resistant *Plasmodium falciparum*. *Biomedical Reports*.

[9] Organization for Economic Cooperation and Development (OECD) (2018). *Guideline 407. Repeated dose 28-day oral toxicity study in rodents*.

[10] Peters W. (1975). The value of drug-resistant strains of *Plasmodium berghei* in screening for blood schizontocidal activity. *Annals of Tropical Medicine and Parasitology*.

[11] Peters W. (1965). Drug resistance in *Plasmodium berghei* Vincke and Lips, 1948. I. Chloroquine resistance. *Experimental Parasitology emphasizes*.

[12] Ryley J., Peters W. (1970). he antimalarial activity of some quinolone esters. *Annals of Tropical Medicine & Parasitology*.

[13] OECD (2001). *OECD guideline for testing of chemicals. Acute oral toxicity–Up-anddown procedure*.

[14] Gorobets N. Y., Sedash Y. V., Singh B. K., Poonam, Rathi B. (2017). An Overview of Currently Available Antimalarials. *Current Topics in Medicinal Chemistry*.

[15] Jansen O., Tchinda A. T., Loua J. (2017). Antiplasmodial activity of Mezoneuron benthamianum leaves and identification of its active constituents. *Journal of Ethnopharmacology*.

[16] Wallqvist A., Fang X., Tewari S. G., Ye P., Reifman J. (2016). Metabolic host responses to malarial infection during the intraerythrocytic developmental cycle. *BMC Systems Biology*.

[17] Omoregie E. S., Pal A. (2016). Antiplasmodial, antioxidant and immunomodulatory activities of ethanol extract of *Vernonia amygdalina* del. Leaf in Swiss mice. *Avicenna Journal of Phytomedicine*.

[18] Tripathy S., Roy S. (2015). Redox sensing and signaling by malaria parasite in vertebrate host. *Journal of Basic Microbiology*.

[19] Vasconcellos L. R., Dutra F. F., Siqueira M. S. (2016). Protein aggregation as a cellular response to oxidative stress induced by heme and iron. *Proceedings of the National Acadamy of Sciences of the United States of America*.

[20] Schwarzer E., Arese P., Skorokhod O. A. (2015). Role of the lipoperoxidation product 4-hydroxynonenal in the pathogenesis of severe malaria anemia and malaria immunodepression. *Oxidative Medicine and Cellular Longevity*.

[21] Suchal K., Malik S., Gamad N. (2016). Kaempferol attenuates myocardial ischemic injury via inhibition of MAPK signaling pathway in experimental model of myocardial ischemia-reperfusion injury. *Oxidative Medicine and Cellular Longevity*.

[22] Suchal K., Malik S., Gamad N. (2016). Kampeferol protects against oxidative stress and apoptotic damage in experimental model of isoproterenol-induced cardiac toxicity in rats. *Phytomedicine*.

[23] Al-Numair K. S., Chandramohan G., Veeramani C., Alsaif M. A. (2015). Ameliorative effect of kaempferol, a flavonoid, on oxidative stress in streptozotocin-induced diabetic rats. *Redox Report*.

[24] Wong S. K., Jann M. L. S., Sudi S. (2015). Anti-malarial and anti-inflammatory effects of gynura procumbens are mediated by kaempferol via inhibition of glycogen synthase kinase-3*β* (GSK3*β*). *Sains Malaysiana*.

[25] Wang H., Kumar A., Lamont R. J., Scott D. A. (2014). GSK3beta and the control of infectious bacterial diseases. *Trends in Microbiology*.

[26] Kim T. W., Lee S. Y., Kim M., Cheon C., Ko S. (2018). Kaempferol induces autophagic cell death via IRE1-JNK-CHOP pathway and inhibition of G9a in gastric cancer cells. *Cell Death & Disease*.

[27] Qiu W., Lin J., Zhu Y. (2017). Kaempferol modulates DNA methylation and downregulates DNMT3B in bladder cancer. *Cellular Physiology and Biochemistry*.

[28] Lee J., Kim J. H. (2016). Kaempferol inhibits pancreatic cancer cell growth and migration through the blockade of EGFR-related pathway in vitro. *PLoS ONE*.

[29] Das A. (2015). Anticancer effect of antimalarial artemisinin compounds. *Annals of Medical and Health Sciences Research*.

[30] Du Plessis L. H., Govender K., Denti P., Wiesner L. (2015). In vivo efficacy and bioavailability of lumefantrine: Evaluating the application of Pheroid technology. *European Journal of Pharmaceutics and Biopharmaceutics*.

[31] Anosa G. N., Udegbunam R. I., Okoro J. O., Okoroafor O. N. (2014). In vivo antimalarial activities of Enantia polycarpa stem bark against Plasmodium berghei berghei in mice. *Journal of Ethnopharmacology*.

